# Sacral Tumours on MRI: A Pictorial Essay

**DOI:** 10.5334/jbsr.1887

**Published:** 2019-11-08

**Authors:** Eva Vanheule, Wouter Huysse, Nele Herregods, Koenraad Verstraete, Lennart Jans

**Affiliations:** 1 Ghent University, BE

**Keywords:** Sacrum, Bone tumour, MRI, musculoskeletal imaging

## Abstract

Tumours of the sacrum can be primary or secondary. Since the sacrum is rich in haematopoietic bone marrow, bone metastases are the most frequent aetiologies. However, tumours can arise from all components of the sacrum and primary bone tumours should be considered in case of a solitary lesion and absence of oncologic history. As the clinical signs are usually non-specific, magnetic resonance imaging has become an indispensable tool in narrowing the differential diagnosis and determining the therapeutic approach. This pictorial essay illustrates specific features of the most common sacral tumours on magnetic resonance (MR) imaging.

## Introduction

Metastases are the most common tumours in the sacrum. However, all components of the sacrum can give rise to benign or malignant tumours and some primary bone tumours present a particular predilection for the sacrum, especially chordoma and giant cell tumour of bone [1,2]. Magnetic resonance (MR) imaging helps narrowing the differential diagnosis and plays an important role in further management of the patient [2,3]. This pictorial review highlights the specific imaging features of sacral tumours on MRI.

## Primary Tumours

**Figure 1:**
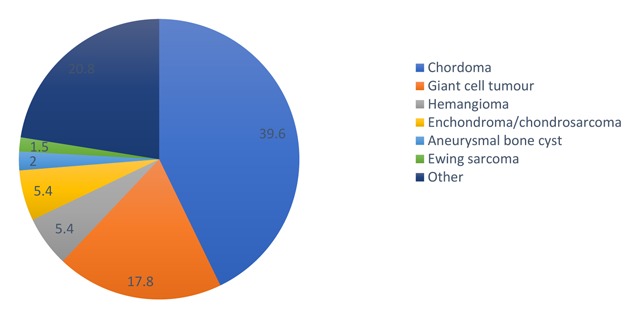
Prevalence of primary sacral bone tumours.

Approximately 5% of the sacral tumours are primary tumours [4]. A detailed overview of the distribution of these tumours is given in Figure 1 [5].

### Chordoma

**Figure 2:**
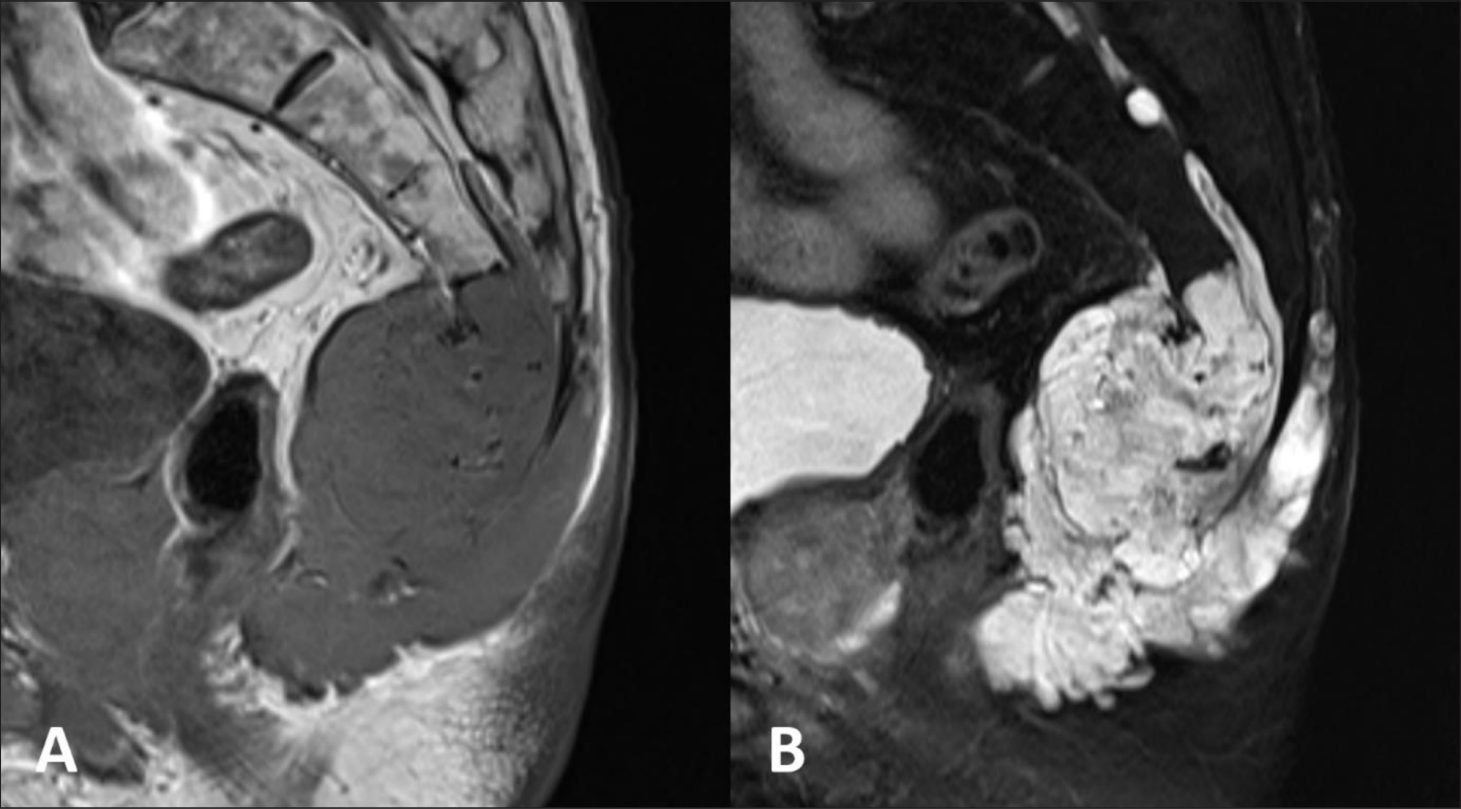
A 79-year-old male with sacral chordoma. **(a)** Sagittal T1-weighted MR image shows a hypointense mass arising from the lower sacrum with a large soft tissue component. **(b)** Sagittal T2-weighted MR image shows a lobulated mass with a heterogeneous hyperintense signal, invading the bone and soft tissues.

Chordomas are the most common primary sacral tumours, accounting for almost 40% [2,3]. The highest incidence is noted in the fifth and sixth decades, with a male-to-female ratio of 2:1. Derived from notochord remnants, the malignant tumour has a predilection for sacral locations and often develops midline or paramedian, with posterior extension and soft tissue invasion [6]. On MRI, the lesions appear as a lobulated mass with heterogeneous hypo- to isointense signal on T1-weighed images, hyperintense signal on T2-weighted sequences (Figure 2). Moderate enhancement is observed after gadolinium injection. Hyperintense foci on T1-weighted images indicates either haemorrhage or proteinaceous content, while hypointense signal on T2-weighted images should be interpreted as old haemorrhage or septations [1,6,7].

### Giant cell tumour

**Figure 3:**
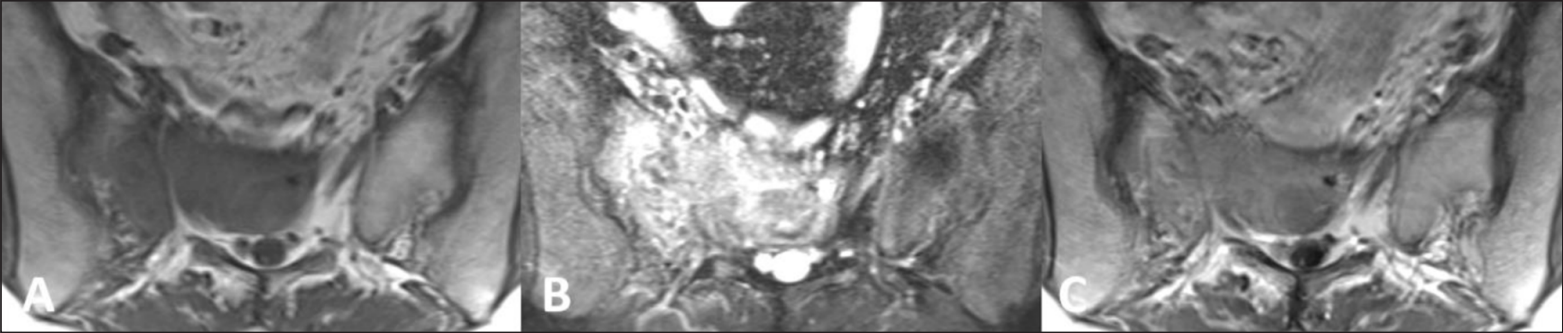
A 40-year-old female with giant cell tumour of the sacrum. **(a)** Axial T1-weighted MR image shows a homogeneous hypointense mass located on both sides of the midline with extension into the presacral soft tissues. **(b)** Axial fat saturated T2-weighted MR image shows heterogeneous high signal intensity. **(c)** Axial post-contrast T1-weighted MR image shows moderate enhancement.

Giant cell tumour of bone, also known as osteoclastoma, is the second most common primary tumour in the sacrum [5]. Patients aged 15–50 are most frequently affected, and women are twice as likely to develop the tumour compared to men [3,8]. Giant cell tumours are usually located on both sides of the midline and extension across the sacroiliac joint is frequent (Figure 3). The solid components show a low to intermediate signal on T1-weighted images and usually enhance intensely after administration of gadolinium. The heterogeneous signal on T2-weighted sequences is variable and non-specific. Cystic changes and fluid-fluid levels are occasionally observed [8].

### Haemangioma

**Figure 4:**
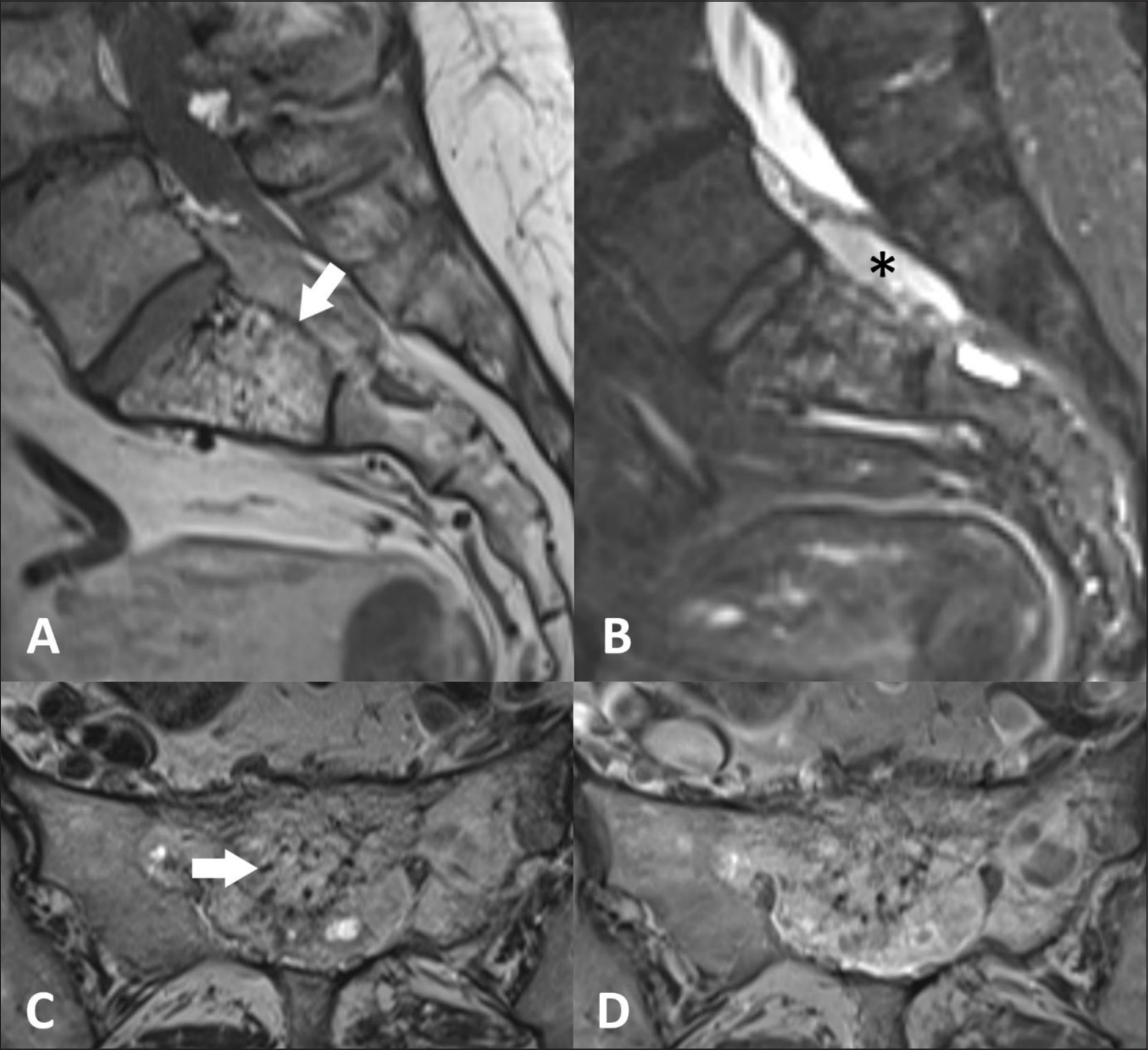
A 65-year-old female with sacral hemangioma. **(a)** Sagittal T1-weighted MR image and **(b)** sagittal fat saturated T2-weighted MR image shows a S1 sacral hemangioma with an atypical extra-osseous component extending into the spinal canal (asterix). Note the maintained vertebral height and cortical integrity (arrow). **(c)** Axial T2-weighted MR image shows hyperintense signal with multiple punctuate hypointense areas (polka-dot sign) (arrow). **(d)** Axial post-contrast T1-weighted MR image shows heterogeneous enhancement of the tumour.

Although vertebral haemangiomas occur in more than 11% of the population, sacral involvement is rare. The tumour is most prevalent during the fifth decade, with a male to female ratio of 1:1.5. Typical haemangiomas are fat predominant, creating a hyperintense well-defined signal on T1- and T2-weighted images. Due to the presence of vascular elements the signal remains high on fluid-sensitive sequences. Linear areas of lower signal correspond to thickened vertical trabeculae and are referred to as the polka dot sign (Figure 4). Atypical haemangiomas are composed of less fatty and more vascular components creating a hypointense to isointense signal on T1-weighted images with variable enhancement and heterogeneous hyperintense signal on both T2-weighted images and STIR-sequences. As a result the polka dot sign is more difficult to distinguish. Maintained vertebral height, a sharp margin, intact bone cortex and enlarged paraspinal vessels are additional findings that favour the diagnosis of haemangioma [9].

### Enchondroma/Chondrosarcoma

**Figure 5:**
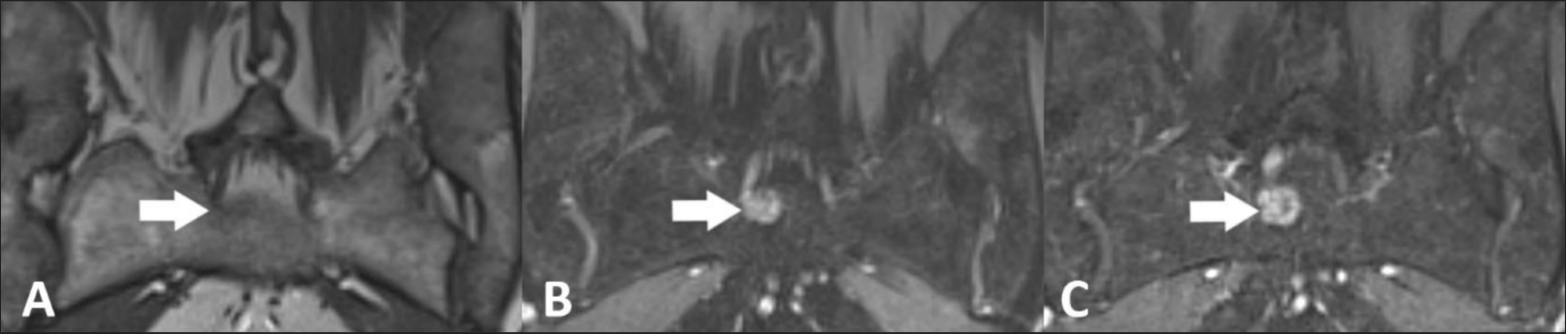
A 51-year-old male with small sacral enchondroma (arrow). **(a)** Coronal T1-weighted MR image shows a hypointense mass in the body of the sacrum. **(b)** Coronal fat-saturated T2-weighted MR image shows a lobulated hyperintense mass. Note absence of bone marrow oedema. **(c)** Coronal fat-saturated post-contrast T1-weighted MR image shows septal enhancement.

Enchondromas originate from the medullary cavity and are particularly common in long bones. Sacral location is rare. They are most often diagnosed during the fourth and fifth decade of life with an equal gender distribution [10]. On MRI, the lobules of firm matured cartilage create a hypointense to isointense signal on T1-weighted sequences and a hyperintense signal on T2-weighted sequences. Regions of calcification and osseous content translate as a low signal on T2-weighted images with either high, intermediate or low signal on T1-weighted images. Enhancement along the fibrovascular septa surrounding the cartilaginous lobules may be observed after administration of gadolinium [11] (Figure 5). Bone or soft tissue oedema are rare, as well as soft tissue masses, cortical destruction or solid enhancement. When present, these findings are highly suggestive of chondrosarcoma and a biopsy should be performed [12].

### Aneurysmal bone cyst

**Figure 6:**
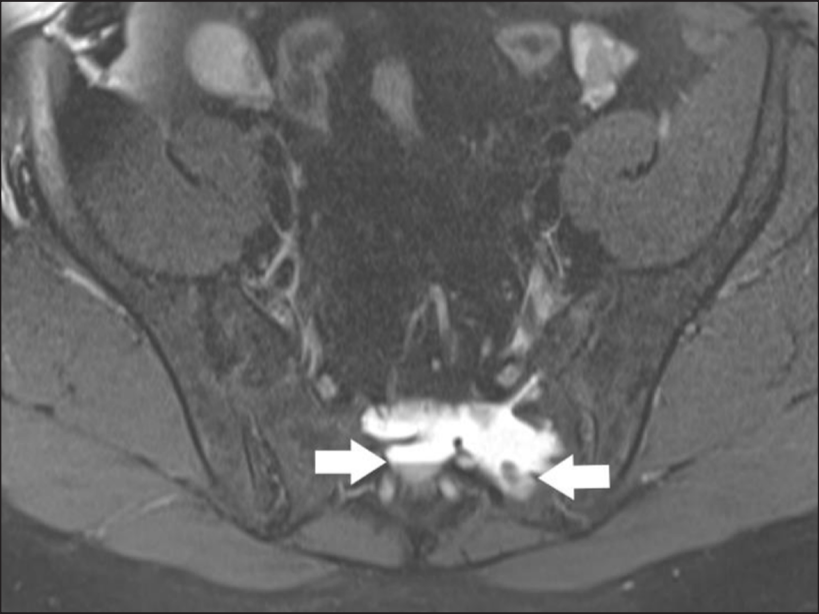
A 43-year-old female with aneurysmal bone cyst. Axial fat saturated T2-weighted MR image shows a lobulated cyst with multiple fluid-fluid levels (arrow). Remark the sedimentation of blood cells caused by slow flow of the blood in the cystic spaces of the tumour.

Aneurysmal bone cysts are primarily seen in children and adolescents with a slight female predominance (56%) [2]. The multiseptated lesion has a heterogeneous appearance on T1- and T2-weighted sequences with a low-intensity rim in the periphery, indicating a thin shell of bone. The typical fluid-fluid levels are diagnostic [13] (Figure 6). As there are usually no solid structures within the cysts, only the septations enhance on T1Gd-sequences [2]. When solid components are present, the MRI appearance is very similar to that of a giant cell tumour, albeit with a heterogeneous signal intensity on both T1- and T2-weighted images.

### Ewing sarcoma

**Figure 7:**
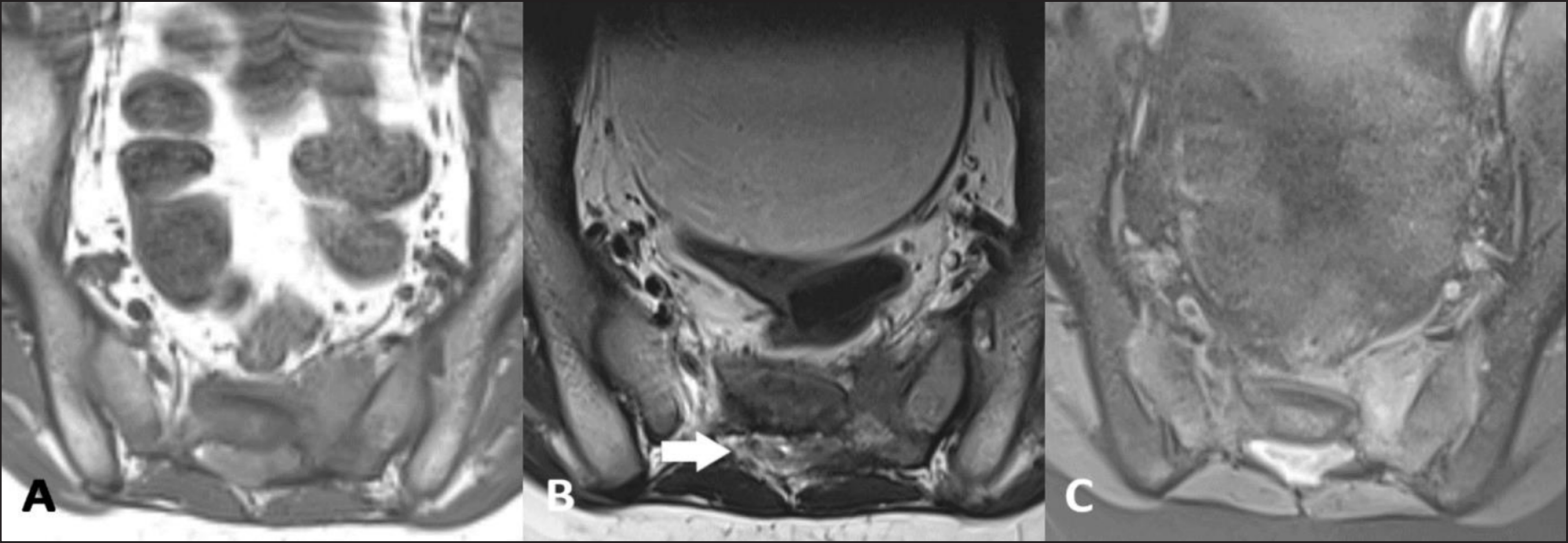
A 14-year-old male with sacral Ewing sarcoma. **(a)** Axial T1-weighted MR image shows a hypointense mass arising from left side of the sacral body. **(b)** Axial T2-weighted MR image shows a large soft-tissue mass, invading into the spinal canal (arrow) and presacral area. Note the absence of hyperintense cerebrospinal fluid signal. **(c)** Axial fat saturated post-contrast T1-weighted MR image shows enhancement of the tumour and the intraspinal component.

Ewing sarcoma is a high-grade malignancy composed of uniform small round blue cells. It occurs most frequently during the teenage years, with a male predominance of 62% [1]. The tumour often presents as a destructive osteolytic lesion with a soft tissue component. The lesion has a homogeneous hypo- to isointense signal on T1-weighted images and isointense signal on T2-weighted images. The extraosseous component is typically larger than the intraosseous lesions and invasion into the paraspinal area and spinal cord is common (Figure 7). After the administration of gadolinium mild to moderate enhancement is observed [1,14].

## Metastases

**Figure 8:**
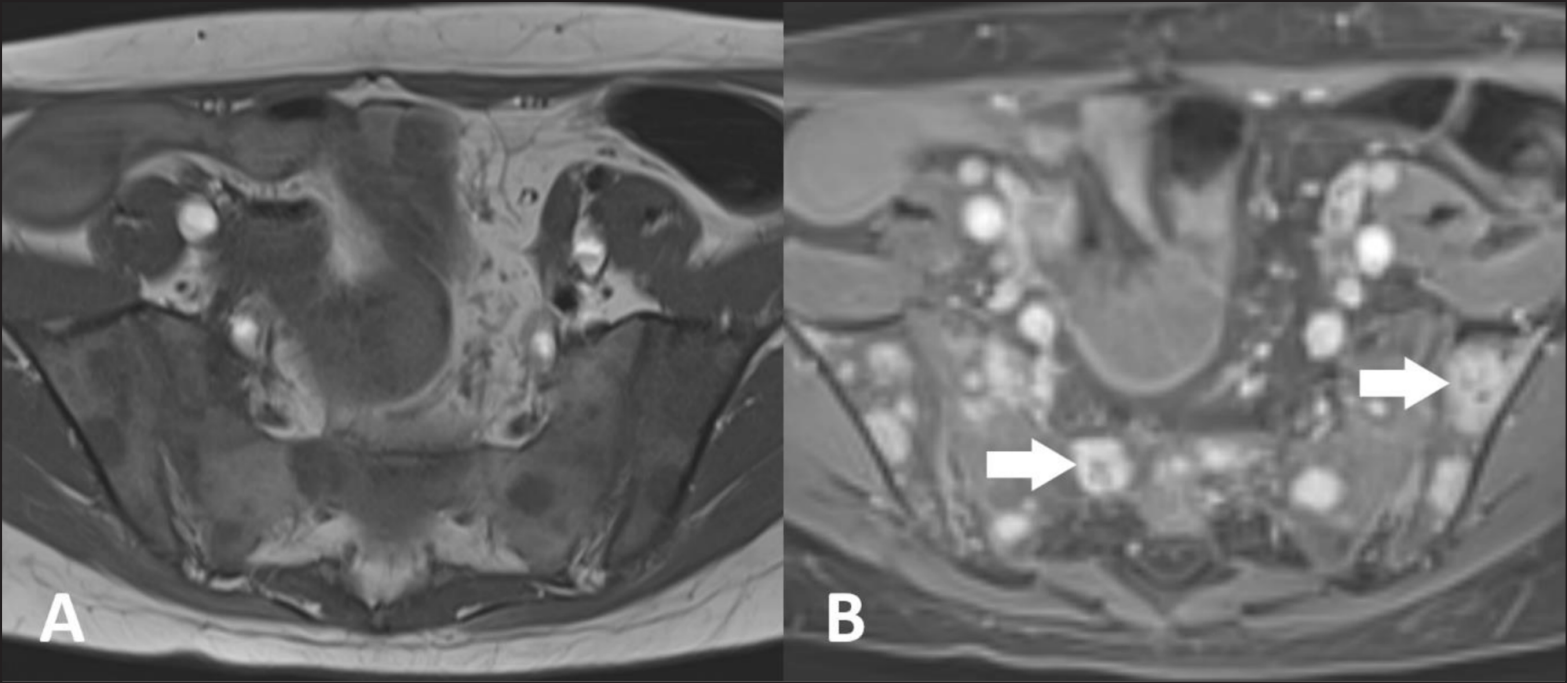
A 13-year-old female with hemangiosarcoma and sacral metastasis. **(a)** Axial T1-weighted MR-image shows multiple hypointense lesions in sacral and iliac bone. **(b)** Axial fat-saturated post-contrast T1-weighted MR image shows strong enhancement of the lesions with central necrosis in the largest lesions (arrow).

Lung, breast, prostate, kidney, head and neck, skin (melanoma) and gastro-intestinal cancers are the most common tumours that can produce sacral metastases. Most metastases are osteolytic except for prostate cancer, where metastases are mainly osteoblastic [1]. Osteolytic lesions have a hypointense signal on T1-weighted sequences and an iso- to hyperintense signal on T2-weighted sequences compared to normal bone marrow (Figure 8). On STIR sequences the signal is usually hyperintense. Administration of gadolinium most often leads to intense uptake although moderate, heterogeneous and low uptake are not uncommon. A peripheral hyperintense rim on T2-weighted images is a rather rare but highly specific finding. Cortical disruption is seen in 57% of all osteolytic metastases. In osteosclerotic metastases, the sclerotic areas appear hypointense on all sequences [15,16].

## Conclusions

The differential diagnosis of sacral tumours is extensive, and although metastases are the most common lesions, a broad spectrum of primary bone tumours can arise from sacral components. MR imaging allows us to detect bone marrow invasion, and changes in morphology and intensity will help to determine the most probable diagnosis. In addition, imaging has an important role in the staging of the tumour and further follow-up.

## References

[1] Thornton, E, Krajewski, KM, O’Regan, KN, Giardino, AA, Jagannathan, JP and Ramaiya, N. Imaging features of primary and secondary malignant tumours of the sacrum. Br J Radiol. 2012; 85(1011): 279–86. DOI: 10.1259/bjr/25247602 22167504 PMC3473982

[2] Gerber, S, Ollivier, L, Leclere, J, et al. Imaging of sacral tumours. Skeletal Radiol. 2008; 37(4): 277–89. DOI: 10.1007/s00256-007-0413-4 18034341

[3] Llauger, J, Palmer, J, Amores, S, Bague, S and Camins, A. Primary tumors of the sacrum: Diagnostic imaging. AJR Am J Roentgenol. 2000; 174(2): 417–24. DOI: 10.2214/ajr.174.2.1740417 10658718

[4] Ong, KO and Ritchie, DA. Pictorial essay: Tumours and pseudotumours of sacrum. Can Assoc Radiol J. 2014; 65(2): 113–20. DOI: 10.1016/j.carj.2013.05.002 24125655

[5] Zhou, Z, Wang, X, Wu, Z, Huang, W and Xiao, J. Epidemiological characteristics of primary spinal osseous tumors in Eastern China. World J Surg Oncol. 2017; 15(1): 73 DOI: 10.1186/s12957-017-1136-1 28376922 PMC5379532

[6] Pillai, S and Govender, S. Sacral chordoma: A review of literature. J Orthop. 2018; 15(2): 679–84. DOI: 10.1016/j.jor.2018.04.001 29881220 PMC5990241

[7] Behaeghe, M, Denis, A, Jans, L and Verstraete, K. Sacral chordoma. Jbr-btr. 2013; 96(1): 51 DOI: 10.5334/jbr-btr.204 23610894

[8] Kwon, JW, Chung, HW, Cho, EY, et al. MRI findings of giant cell tumors of the spine. AJR Am J Roentgenol. 2007; 189(1): 246–50. DOI: 10.2214/AJR.06.1472 17579178

[9] Gaudino, S, Martucci, M, Colantonio, R, et al. A systematic approach to vertebral hemangioma. Skeletal Radiol. 2015; 44(1): 25–36. DOI: 10.1007/s00256-014-2035-y 25348558

[10] Deckers, C, Schreuder, BH, Hannink, G, de Rooy, JW and van der Geest, IC. Radiologic follow-up of untreated enchondroma and atypical cartilaginous tumors in the long bones. J Surg Oncol. 2016; 114(8): 987–91. DOI: 10.1002/jso.24465 27696436 PMC6222252

[11] Guo, J, Gao, JZ, Guo, LJ, Yin, ZX and He, EX. Large enchondroma of the thoracic spine: a rare case report and review of the literature. BMC Musculoskelet Disord. 2017; 18(1): 155 DOI: 10.1186/s12891-017-1519-z 28407736 PMC5390427

[12] Douis, H, Parry, M, Vaiyapuri, S and Davies, AM. What are the differentiating clinical and MRI-features of enchondromas from low-grade chondrosarcomas? Eur Radiol. 2018; 28(1): 398–409. DOI: 10.1007/s00330-017-4947-0 28695356

[13] Zileli, M, Isik, HS, Ogut, FE, Is, M, Cagli, S and Calli, C. Aneurysmal bone cysts of the spine. Eur Spine J. 2013; 22(3): 593–601. DOI: 10.1007/s00586-012-2510-x 23053752 PMC3585636

[14] Huang, WY, Tan, WL, Geng, DY, et al. Imaging findings of the spinal peripheral Ewing’s sarcoma family of tumours. Clin Radiol. 2014; 69(2): 179–85. DOI: 10.1016/j.crad.2013.09.010 24188594

[15] Shah, LM and Salzman, KL. Imaging of spinal metastatic disease. Int J Surg Oncol. 2011; 2011: 769753 DOI: 10.1155/2011/769753 22312523 PMC3263660

[16] Bratu, AM, Raica, VP, Salcianu, IA, et al. MRI differential diagnosis: bone metastases versus bone lesions due to malignant hemopathies. Rom J Morphol Embryol. 2017; 58(4): 1217–28. 29556610

